# Retinal hemorrhages following fingolimod treatment for multiple sclerosis; a case report

**DOI:** 10.1186/s12886-015-0125-9

**Published:** 2015-10-19

**Authors:** Naoko Ueda, Kyoko Saida

**Affiliations:** Department of Ophthalmology, Kyoto Hakuaikai Hospital, 1 Keshiyama, Kamigamo, Kita-ku, Kyoto, 603-8041 Japan; Department of Neurology, Kyoto Hakuaikai Hospital, 1 Keshiyama, Kamigamo, Kita-ku, Kyoto, 603-8041 Japan

**Keywords:** Retinal hemorrhages, Fingolimod, Relapsing-remitting multiple sclerosis, Side effect, Macular edema

## Abstract

**Background:**

Fingolimod is the first oral agent used for treatment of relapsing-remitting multiple sclerosis. Macular edema, but not retinal hemorrhage, is a well-known adverse effect of fingolimod treatment. To the best of our knowledge, this is the first case report of extensive retinal hemorrhages following fingolimod treatment.

**Case presentation:**

A 31-year-old male with relapsing-remitting multiple sclerosis developed macular edema and retinal hemorrhages in his left eye, 1 month after starting fingolimod treatment; treatment was then discontinued. The hemorrhages were flame-shaped, and were extensive along retinal arteries and veins. The hemorrhages started to decrease at 4 weeks and disappeared completely at 24 weeks after cessation of fingolimod treatment.

**Conclusions:**

Occurrence of retinal hemorrhage warrants careful follow-up for multiple sclerosis patients treated with fingolimod.

## Background

Fingolimod (Gilenya®, Novartis, Emeryville, CA, USA) is the first oral agent used for treatment of relapsing-remitting multiple sclerosis (RRMS). Macular edema (ME) is a well-known adverse effect of fingolimod treatment, occurring in approximately 0.4 % of patients [fingolimod-associated macular edema (FAME)] [1]. However, retinal hemorrhage has been almost unrecognized as a side effect of fingolimod treatment. We therefore report a case of extensive flame-shaped retinal hemorrhages in a patient treated with fingolimod.

## Case presentation

A 31-year-old male with a 13-year history of RRMS, after fingolimod treatment for 1 month, was diagnosed with ME and retinal hemorrhages in his left eye at a regular ophthalmic examination. His past history revealed that the onset of MS was accompanied by a visual disorder. He presented with several gadolinium-enhanced active lesions in his brain, despite therapies of steroid pulse, plasma exchanges, immunoglobulin, and interferon-beta (−β). Although interferon-β was used for a period of 6 months, 6 years prior, it was discontinued because of allergic skin reactions. The symptoms had improved after undergoing a course of five plasma exchanges 5 years prior but the patient refused further treatment. Over several years, the patient had developed paralysis in his right upper limb, both lower limbs, and had severe urinary incontinence and constipation. His Expanded Disability Score Scale (EDSS) was 8.5. Anti-aquaporin-4 antibody tested negative. Laboratory studies, including bleeding and coagulation tests, were within normal limits. He had no history of hypertension (blood pressure approximately 90–105/50–75 mmHg), diabetes mellitus, or hematological diseases. When retinal hemorrhages were recognized, the hemoglobin level was 15.0 g/dL and the platelet count was 210,000/μL (within the normal range). The patient had no history of uveitis and pars planitis.

**Fig. 1:**
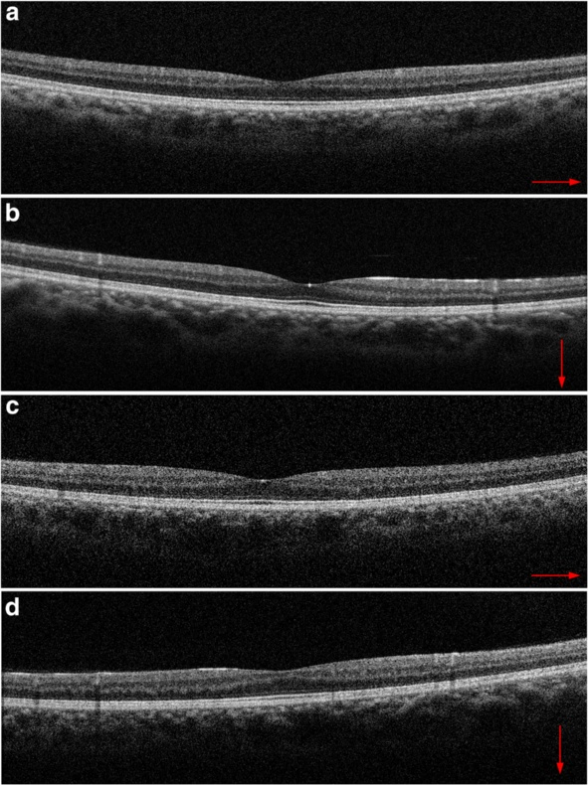
Spectral domain optical coherence tomography (SD-OCT) scans of both eyes before fingolimod treatment. The thinning of retinal nerve fiber layers was recognized, with **(a)** showing horizontal and **(b)** showing vertical images in the right eye, and **(c)** showing horizontal and **(d)** showing vertical images in the left eye. Because of the patient’s nystagmus, the precise averaging of multiple SD-OCT B-scans was not possible, so single B-scan images are shown

At the first ophthalmological examination before taking fingolimod, his corrected visual acuity was 20/600 OD and 20/400 OS, optic disc color was pale, and spectral domain optical coherence tomography (SD-OCT) showed a thinner retina (particularly in the nerve fiber layer) without ME in both eyes (Fig. 1). This was regarded as the cause of decreased visual acuity. In all directions, the patient showed gaze-evoked nystagmus.

**Fig. 2:**
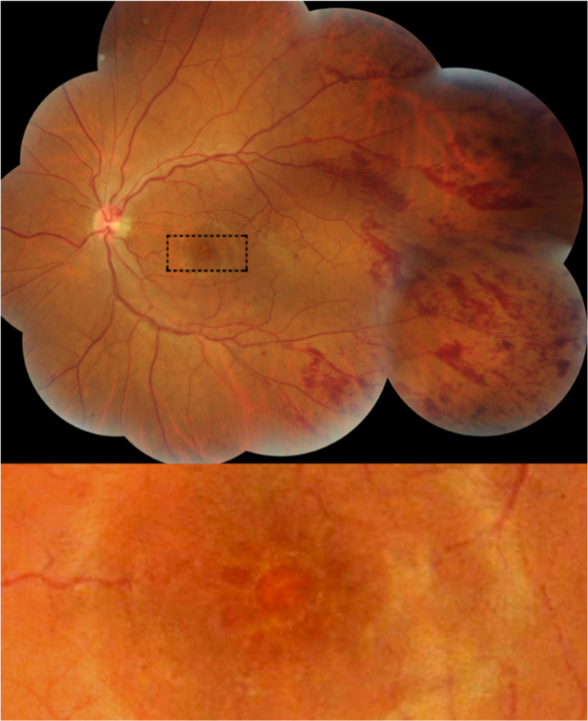
Color fundus photography of the patient. Flame-shaped hemorrhages are seen along the retinal arteries and veins in the left eye 1 month after starting fingolimod treatment. Deeper dot-blot hemorrhages, and a hemorrhage on the disc at the 12 to 1 o’clock position, were also recognized. Moderate macular edema is also shown

**Fig. 3:**
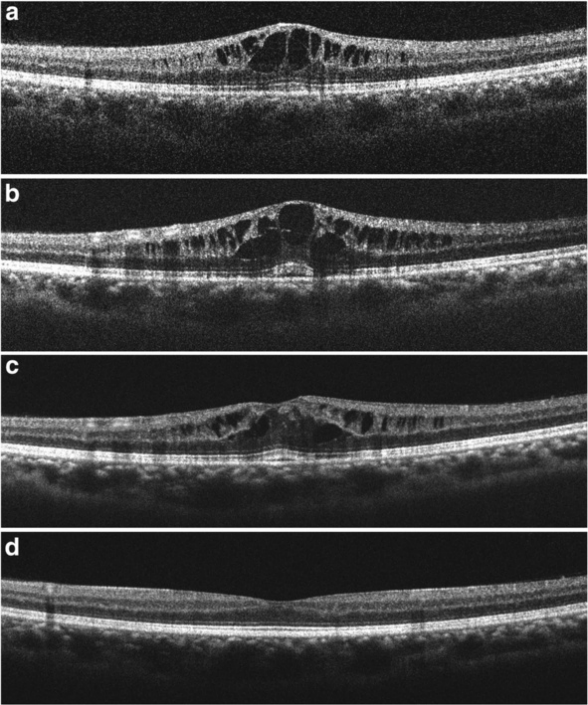
Spectral domain optical coherence tomography (SD-OCT) scans through the fovea. **a** Four weeks after starting fingolimod treatment, SD-OCT showed cystoid macular edema in the left eye. At that time, fingolimod was terminated. **b** Three weeks and **(c)** 13 weeks after cessation of fingolimod treatment, macular edema was still present. Topical betamethasone treatment began at 13 weeks. **d** Macular edema resolved 4 weeks after topical steroid treatment. Because of the patient’s nystagmus, the precise averaging of multiple SDOCT B-scans was not possible, and single B-scan images are shown

Four weeks after fingolimod treatment, his left eye revealed extensive flame-shaped retinal hemorrhages along retinal arteries and veins (Fig. 2) as well as cystic ME, as measured by SD-OCT (Fig. 3). His visual acuity did not decrease considerably, with 20/600 OD and 20/500 OS. Most of the hemorrhages were found along both retinal arteries and veins beyond the mid-periphery involving all four quadrants of the retina. Deeper dot-blot hemorrhages, and a hemorrhage on the optic disc at the 12 to 1 o’clock position, were also recognized. The diameter and tortuosity of the retinal veins after the hemorrhages were the same as before the hemorrhages. Both eyes had no inflammatory signs in the anterior segment and vitreous, as assessed by slit lamp biomicroscopy examination. Fingolimod was discontinued. Because FAME remained for 13 weeks, topical treatment with 0.1 % betamethasone, four times daily, was started. FAME was resolved completely 4 weeks after starting topical steroid therapy; that was 17 weeks after the cessation of fingolimod. Retinal hemorrhages remained unchanged for 4 weeks after the cessation of fingolimod treatment, then started to decrease and disappeared completely at 24 weeks, indicating that the hemorrhages existed for 7 weeks longer than the FAME. During the treatments and follow-ups, neither retinal hemorrhages nor ME developed in the right eye. The patient’s visual acuity at the time of disappearance of retinal hemorrhages and FAME was 20/400 OD and 20/400 OS. Fluorescein angiography was not performed because the patient could not retain a sitting position.

## Discussion

This case study revealed extensive flame-shaped retinal hemorrhages in addition to ME, following fingolimod treatment. The retinal hemorrhages were mainly present at the mid-periphery. There were no differences of retinal vein dilatation and tortuosity before and after hemorrhaging. The hemorrhage pattern was considered to be different from that of central or branch retinal vein occlusion. The patient had no history of hypertension, diabetes mellitus, or hematological diseases. Eales disease and tuberculous vasculitis also cause uni/bilateral peripheral retinal hemorrhages. Although the purified protein derivative (PPD) skin test was not done, the hemorrhages gradually disappeared completely in 24 weeks without oral corticosteroid or anti-tuberculosis treatment after fingolimod was discontinued. There were no signs of vascular occlusion or retinal neovascularization that are often recognized in Eales disease.

To our knowledge, there has been only one report of a macular hemorrhage without apparent causes following treatment with fingolimod [2] that was completely resolved soon after discontinuation of fingolimod. This case report suggests that fingolimod may play a role in disrupting vascular integrity, because hemorrhages are not routinely seen in MS patients without other signs of uveitis.

FAME is a well-known side effect of fingolimod. The sphingosine-1-phosphate (S1P) receptor plays a role in regulating vascular permeability, and enhancing endothelial barrier integrity. Fingolimod, a structural analog of S1P, inhibits this barrier action and leads to increased vascular permeability [3]. This may be the pathophysiological mechanism involving FAME.

Lightman et al. reported that cases of acute optic neuritis are characterized by retinal vascular abnormalities [4]. Their fluorescein angiograms showed multiple site leakage in the mid-peripheral retina. Optic neuritis patients with vascular abnormalities have a tendency to develop MS [4]. Recently, microcystic ME, predominantly affecting the inner nuclear layer, was reported in 4.7 % of patients with MS, and was more common in eyes with higher Multiple Sclerosis Severity Scores [5]. The presence of microcystic ME in MS suggests that there may be a breakdown of the blood-retinal barrier and tight junction integrity [5]. These observations suggest that the mid-peripheral retinal hemorrhages described in the present case report may have been associated with MS-associated uveitis, and could have introduced blood retinal barrier disruption.

In the present case, we found thinning of nerve fiber layers at the same level in both eyes, but it was only in the left eye that ME and retinal hemorrhages were developed. In FAME, 74 % of onset is the single eye onset type [6]. In microcystic edema in MS, two-thirds of the cases are reported as the single eye onset type [5]. The cause of symptom development in only a single eye is unclear, but MS patients are known to develop multiple and asymmetric symptoms.

Our case report suggests that not only multiple sclerosis inflammatory disease, but also MS treatment with fingolimod, may lead to an increase in vascular permeability in some patients. Besides FAME, in severe cases of MS with persistent inflammation, fingolimod may also cause retinal hemorrhage.

## Conclusions

Occurrence of retinal hemorrhages warrants careful follow-up of MS patients treated with fingolimod.

## Consent

Because the patient could not move his hands smoothly, written informed consent was obtained from the patient’s mother for publication of this case report and accompanying images. A copy of the written consent is available for review by the Editor of this journal.

### Ethics approval

Approval for this work was obtained from the Hakuaikai Ethics Committee of Kyoto, Japan.

## Abbreviations

RRMS: Relapsing-remitting multiple sclerosis
ME: Macular edema
FAME: Fingolimod-associated macular edema
EDSS: Expanded Disability Score Scale
SD-OCT: Spectral domain optical coherence tomography
MS: Multiple sclerosis
S1P: Sphingosine-1-phospate
